# Research on Multi-Frequency Vibration Dynamic Compensation Scheme for Electron Beam Inspection Equipment

**DOI:** 10.3390/mi17030336

**Published:** 2026-03-10

**Authors:** Junhai Jiang, David Wei Zhang, Ziyu Liu

**Affiliations:** School of Microelectronics, Fudan University, Shanghai 200433, China; dwzhang@fudan.edu.cn (D.W.Z.); liuziyu@fudan.edu.cn (Z.L.)

**Keywords:** electron beam inspection, single and multi-frequency vibration, positioning information, dynamic compensation, production line application

## Abstract

In the manufacturing process of advanced integrated circuits, electron beam inspection equipment is crucial for yield assurance, while vibration poses a core challenge affecting its precision and speed. Vibrations in production line equipment are mostly multi-frequency; However, research findings in this field remain limited. Moreover, existing compensation schemes often struggle to meet industrial-grade precision and real-time requirements. This paper presents the design and implementation of a high-speed electron beam vibration compensation system based on positioning. The system incorporates state-of-the-art laser positioning and electrostatic scanning deflectors, and features an integrated signal processing and compensation signal output module. The study involved improvements and optimizations to the positioning processing analysis and compensation module, control software and algorithms, and calibration software and algorithms, demonstrating superior performance compared to existing methods. System validation data demonstrates that the proposed scheme effectively compensates for both single-frequency and multi-frequency disturbances at frequencies below 200 Hz, achieving an average attenuation of 50% to 90% and a repetitive compensation accuracy of less than 0.3 nm. These metrics meet the industrial application requirements for electron beam inspection equipment. The overall error in long-term repeatability tests complies with the stability demands of industrial production lines, confirming its practical applicability in production environments.

## 1. Introduction

In the manufacturing of advanced integrated circuits (ICs) [1], the continual scaling of process nodes and increasing structural complexity are driving critical defects toward smaller sizes, higher densities, and greater difficulty of detection, posing a significant threat to production yield. Electron Beam Inspection (EBI), which utilizes scanning electron microscope (SEM) imaging principles, has become indispensable for capturing these defects due to its superior resolution and electrical sensitivity. However, the performance of EBI is critically undermined by mechanical vibration, a core factor limiting both imaging accuracy and operational throughput. Despite its importance, dedicated research on vibration mitigation specifically for EBI systems remains scarce.

Existing approaches can be categorized as either passive or active control. Passive methods, which rely on vibration source isolation, offer limited efficacy in complex industrial environments. Active compensation schemes, while demonstrating progress, are still plagued by key shortcomings in model adaptability, real-time performance, and compensation precision. For instance, image-based post-processing methods lack real-time capability, and compensation schemes employing mechanical actuators typically introduce millisecond-level latencies and are often restricted to single-frequency compensation below 20 Hz. These limitations directly degrade system throughput and fail to meet the stringent demands of modern IC production lines.

Kwang Oh Jung et al. developed a phase-control method to reduce vibration-induced distortion in SEM images [2]. They observed that high-magnification imaging introduces serrated edge artifacts due to vibration. By using accelerometers to measure vibration frequency, the primary source was attributed to a rotary pump. Their solution involved generating an inverse signal from the frequency analysis of image edge data and applying it to the scanning circuit. Simulations and experiments confirmed that this approach substantially mitigated the jagged edges, thereby improving image quality.

In an experimental study, Yun-Ho Shin et al. aimed to enhance the vibration performance of an SEM system [3]. Through accelerometer measurements and impact hammer excitation, they characterized the system’s frequency response and natural frequencies, identifying horizontal vibration as the most critical. Implemented improvements included isolating the SEM from ancillary computers, reinforcing the base support, optimizing elastic mounts for environmental vibration, and modifying internal isolation mounts to dampen pump-transmitted vibration. These modifications were validated both numerically and experimentally.

Focusing on distortion analysis, M. Pluska et al. introduced a method to disentangle the effects of magnetic field distortion from electrical signal distortion in SEM imaging [4]. This was achieved by recording distorted images at varying working distances and electron energies, followed by distortion measurement, quadratic polynomial fitting, and coefficient analysis. This process allowed them to separate the contributions of different distortion sources and quantify electron beam deflection through image deformation measurements.

Koichi Matsuda’s team proposed an image-processing-based active vibration suppression technique [5,6]. Their method involved analyzing sample images and applying mathematical models with least-squares matching to estimate the electron probe’s pointing error. Subsequently, a digital filter controller was designed based on a transfer function model that related this error to acceleration signals. This controller drives the scanning coils to adjust the probe position in real time, providing effective compensation within the low-frequency band, including the system’s first four natural frequencies.

In the domain of image analysis, You-Jin Park et al. presented an analytical method to quantify the impact of electromagnetic interference (EMI) on semiconductor SEM image distortion [7]. Their approach applies an optimal sequence of filters (Gaussian, Scharr x, and Canny) for denoising and edge detection on EMI-affected images, followed by the extraction of five shape metrics (e.g., object area, contour perimeter). Statistical analysis showed these metrics effectively quantify distortion severity, with values highest under strong EMI and decreasing with weaker interference. The inter-group differences were statistically significant (*p*-value ≈ 0), confirming the method’s efficacy.

For drift correction, Iago Bischoff Montenegro et al. reported a post-processing algorithm for SEM nanoparticle imaging [8]. It generates a high-quality composite image by overlaying multiple rapidly acquired, low-quality, drift-free frames using redundant cross-correlation. For platinum nanoparticles (300–1000 nm), the algorithm improved the mean signal-to-noise ratio (SNR) from 4.4 dB (single image) to 11.3 dB (five overlayed images). Its capability to preserve geometric shape and surface features was verified by atomic force microscopy (AFM), offering a valuable tool for SEM systems lacking hardware drift correction.

Jieping Ding et al. proposed a novel method to quantify and correct inherent distortion in SEM images [9]. The core methodology entails developing an external scan controller, adopting a snake scanning strategy, and leveraging the resultant odd-even row misalignment to segment images into sub-images. Feature points are then extracted via the ORB algorithm to measure pixel offsets for compensation. The key findings indicate that distortion amplitude is primarily governed by dwell time (independent of magnification) and exhibits a stable threshold. Post-correction, image structural similarity and quality metrics are significantly improved, providing a quantifiable solution for SEM and other microscopic techniques.

Additionally, other researchers such as Koichi Matsuda, Damazo, Peter A. Jennings, and Marcelo Gaudenzi de Faria have contributed various methods and insights concerning vibration source analysis, modeling, compensation, and simulation [10,11,12,13].

Research on vibration compensation for large-scale, industrial equipment such as Electron Beam Inspection (EBI) systems remains scarce, creating a significant gap in this niche field. EBI and similar metrology tools possess distinct challenges: their large vacuum chambers are prone to greater stress and deformation, with larger component contact areas than conventional scanning electron microscopes (SEMs). Additionally, their stages feature higher load capacities and more complex mechanical structures, which exacerbate vibration issues.

A key limitation of many existing vibration compensation schemes is their inability to meet the stringent real-time requirements of industrial production. For instance, the image-based post-processing methods employed by Kwang Oh Jung and Koichi Matsuda’s teams necessitate analyzing captured images before applying corrective signals, a process that inherently precludes real-time operation.

Furthermore, compensation approaches that rely on vibration positioning information (e.g., the method by Yun-Ho Shin), while incorporating multi-dimensional sensors, are hindered by low bandwidth and millisecond-level latencies. These methods typically support compensation only for frequencies below 20 Hz, which is insufficient for the high-speed, real-time demands of EBI and metrology equipment on production lines.

To overcome these limitations, the scheme presented in this work integrates advanced technologies encompassing precision laser positioning, motion control, computational prediction, and high-speed control of the electron beam path. It proposes an innovative solution to the prevalent industry problem of multi-frequency vibration interference in optical/electron beam imaging, offering superior compensation accuracy and significantly reduced latency compared to existing state-of-the-art methods.

## 2. Compensation Scheme Design Principles

### 2.1. Impact of Vibration on Electron Beam Imaging and Compensation Capability Range

In practical applications of electron beam measurement or inspection equipment, such as scanning electron microscopes (SEMs), imaging accuracy and measurement stability are significantly challenged by various vibrational interferences. Vibration sources can be categorized into two main types: external transmission and internal generation. External vibrations are primarily transmitted through the foundation and building structure. Among these, ground-borne vibrations from sources such as traffic (e.g., vehicles, subways, ~1–30 Hz), construction activities (~1–20 Hz), and human footsteps (~1–10 Hz) are predominantly low-frequency. Vibrations induced by the operation of other equipment, airflow from HVAC (Heating, Ventilation and Air Conditioning) systems, and acoustic noise within the facility generally exhibit a broader frequency band, often spanning from tens to hundreds of hertz. Internal vibrations originate from the equipment’s own components: coolant flow and stage start-stop motions typically generate low-frequency vibrations (usually <100 Hz); vacuum molecular pumps produce noticeable mid-frequency vibrations due to high-speed rotor rotation, typically in the range of approximately 400–800 Hz; while micro-motions of electromagnetic components and circuit noise within the electron optical system can induce high-frequency vibrations up to several thousand hertz. In terms of impact mechanisms, low-frequency vibrations tend to cause image drift and alignment errors, mid-frequency vibrations directly interfere with electron beam focusing and scanning stability, and high-frequency vibrations degrade the signal-to-noise ratio. In practical engineering, conducting on-site vibration spectrum testing is essential to accurately identify the dominant vibration sources and their characteristic frequencies, thereby enabling the implementation of targeted vibration compensation designs.

Existing Electron Beam Inspection (EBI) and metrology equipment leverage scanning electron microscope (SEM) imaging principles and operate in a raster scan mode. In this mode, the electron beam sequentially scans along the fast-scan direction (typically the X-axis) with a fixed step size (defining the pixel size). Upon completing one line, the beam is deflected back to the beginning of the next line, offset by one step in the slow-scan direction (typically the Y-axis). Given that the beam spot is typically circular and isotropic, the pixel sizes in the X and Y directions are usually set equal.

**Figure 1:**
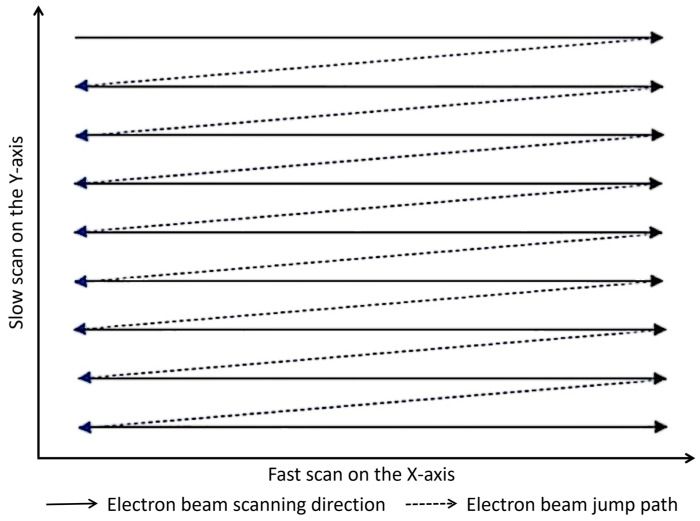
Schematic diagram of the electron beam scanning mode.

Figure 1 illustrates the electron beam scanning mode employed in Electron Beam Inspection (EBI) equipment. In this scheme, the fast-scan direction corresponds to the line scan, while the slow-scan direction corresponds to the frame scan.

Under typical inspection conditions, the fast-scan operates at high speed. For instance, with a pixel dwell time of 100 ns, scanning a line of 1024 pixels requires approximately 100 µs. In contrast, the slow-scan, which comprises multiple sequential line scans, is considerably slower. Acquiring a full frame of 1024 lines thus takes about 100 ms.

Relevant imaging parameters for EBI/metrology equipment and their associated time- and frequency-domain characteristics are summarized in Table 1.

**Figure 2:**
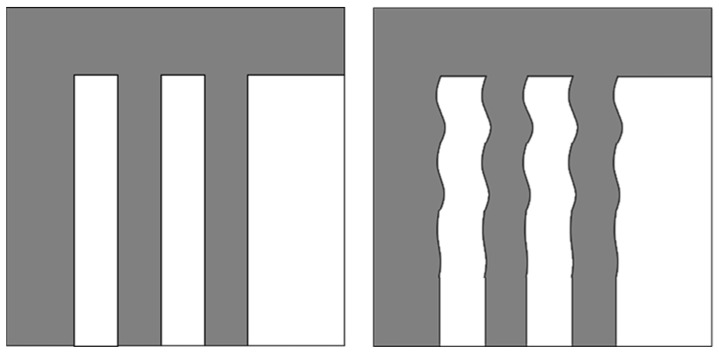
Schematic diagram of the manifestation of vibration in electron beam imaging.

Table 1 indicates that under typical operating conditions, the frame frequency ranges from 10 Hz to 100 Hz. Any mechanical vibration with a frequency higher than this frame rate will induce periodic positional deviations along the X-direction in the acquired image. This effect is illustrated in Figure 2, where a distortion-free image (left) is contrasted with one exhibiting a distinct wavy pattern (right) due to such vibrations.

In practice, multi-frame averaging is commonly employed to suppress detector/circuit noise and improve the signal-to-noise ratio (SNR). However, mechanical vibrations with frequencies lower than the frame rate induce consistent inter-frame offsets, which cannot be mitigated by averaging and thereby directly degrade image quality.

Therefore, based on practical experience and modal analysis, the compensation scheme focuses on vibration signals below 200 Hz, aiming to correct the displacement incurred during each line scan in the fast-scan process. Consequently, the general requirements for electron beam vibration compensation are: (1) to target vibrations below 200 Hz, and (2) to ensure the compensation signal bandwidth exceeds the line frequency (e.g., >100 kHz).

### 2.2. Real-Time Measurement of Vibration Deviation

High-precision measurement of vibrational displacement is fundamental to real-time compensation, placing stringent demands on both the accuracy and temporal resolution of the positioning system. Laser interferometers, widely employed in industrial metrology, are capable of providing sub-nanometer spatial resolution and microsecond-level temporal sampling.

Positioning accuracy is influenced by several factors, including the intrinsic accuracy of the sensor (laser interferometer), the selection of the reference point, and environmental perturbations such as temperature variations and electromagnetic interference (EMI).

Given that system components are not ideally rigid, achieving precise positioning necessitates the calibration of elements on both sides of non-rigid connections. Key calibration targets encompass the stage mirrors and components proximal to the electron optical axis. Accordingly, mirrors were integrated into the experimental setup, on the main chamber top plate and near the objective lens-each with independent measurement paths.

The real-time performance of the positioning system is governed by the sampling rate of the laser interferometer and its controller. In this work, a sampling rate of 10 MHz is utilized. This rate is substantially higher than the required compensation bandwidth (<200 Hz), thereby guaranteeing real-time signal acquisition for effective compensation.

### 2.3. Quantitative Analysis of Compensation Capability

The calculation of compensation values is derived from the scanning imaging model. In an SEM, the primary electron beam is deflected onto the sample, generating secondary and backscattered electrons that are detected at a fixed rate. Each sampling point corresponds to a pixel, whose spatial coordinates are determined by the beam position and whose grayscale value is determined by the detected signal intensity. A rectangular array of these pixels constitutes a frame, which defines the field of view (FOV).

To improve the signal-to-noise ratio (SNR), EBI equipment typically employs signal averaging techniques: pixel averaging (increasing the dwell time per pixel), line averaging (averaging multiple scans of the same line), and frame averaging (averaging multiple frames of the same FOV).

Furthermore, due to the operational characteristics of the deflector drive circuitry, the ideal sawtooth drive waveform exhibits distortion near its minimum and maximum amplitude points. Consequently, signal sampling is confined to the central, linear region of the waveform. The periods at the beginning and end of each line scan—termed the start-line and end-line times—are therefore excluded from sampling.

**Figure 3:**
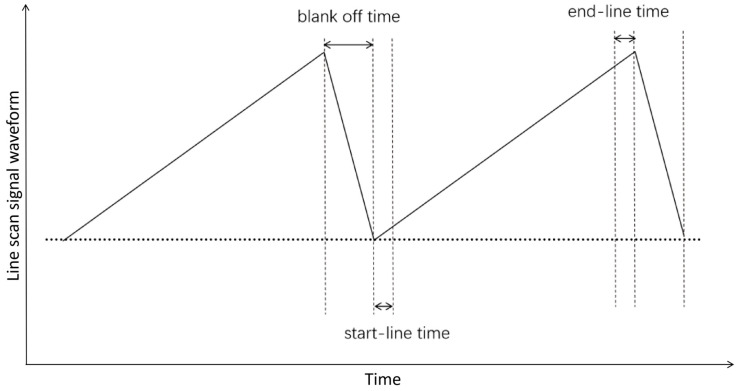
Schematic diagram of the line scan signal.

Following each line scan, the beam blanker deflects the beam away during the retrace period (blank-off time) before the subsequent line begins, which also constitutes a non-sampling interval. The temporal locations of all these non-sampling periods within the line scan signal waveform are illustrated in Figure 3.

The total non-sampling time percentage is small. To increase imaging speed, efforts are made to reduce these times, requiring higher-frequency waveform generation and deflection drive circuits.

The specific electron beam compensation value is closely related to this process. The beam coordinate for a given line is related to the line scan time by:

$$x(t)=x_{0}+{\mathrm{px}}_{x}*(t-t_{0}-t_{\mathrm{SL}})/t_{\mathrm{px}}$$

where $x_{0}$ is the line start coordinate, $t_{0}$ is the line scan start time, $t_{\mathrm{SL}}$ is the start-line time, $t_{\mathrm{px}}$ is the pixel dwell time, and ${\mathrm{px}}_{x}$ is the pixel size.

As indicated in Table 1, the frame frequencies (10–100 Hz) are insufficient to cover the full spectrum of mechanical vibrations. Consequently, compensation must leverage the higher-frequency line scan. This approach is justified because the observed vibration-induced image distortion is consistent along the fast-scan direction. Thus, the compensation scheme primarily employs line compensation (fast-scan), with supplementary column compensation (slow-scan).

When the laser interferometer captures the stage position, the coordinates are recorded and transmitted to calculate the positioning error relative to the expected coordinates. In the step-and-scan mode used, the stage remains relatively stationary, which significantly simplifies the deviation calculation.

## 3. Compensation Scheme Design and Implementation

### 3.1. Experimental Setup

**Figure 4:**
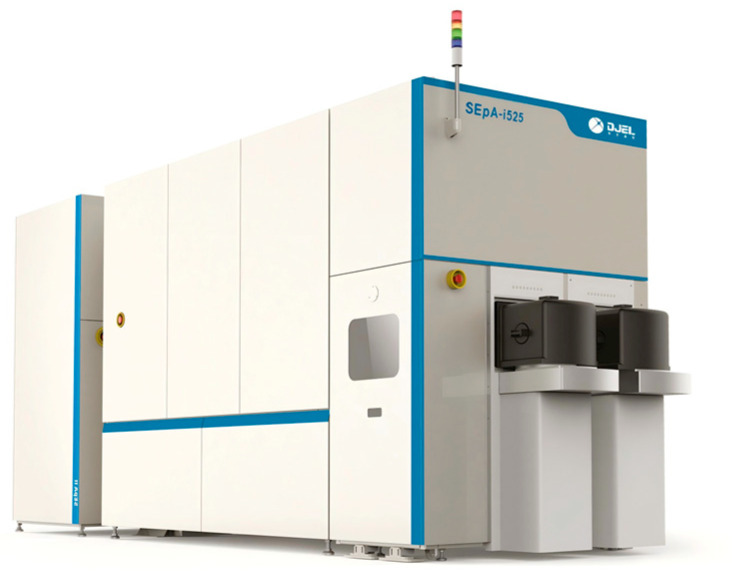
Dongfang Jingyuan SEpA-i525 EBI.

The experiments were conducted using Dongfang Jingyuan’s SEpA-i525 electron beam inspection (EBI) system (Dongfang Jingyuan Electron Co., Ltd., Beijing, China), as shown in Figure 4. This equipment, based on scanning electron microscope (SEM) technology, is designed for direct wafer pattern inspection in front-end integrated circuit (IC) manufacturing. A focused electron beam scans the wafer surface, and the resulting secondary and backscattered electrons are detected and converted into grayscale images that convey both topographical and electrical property information. Defects are identified by comparing image contrasts between different regions on the wafer.

In contrast to optical inspection, EBI provides superior resolution for identifying physical defects and possesses the unique capability to detect electrical defects, such as open circuits and leakage currents.

To assess the vibrational characteristics of the complete machine and its key components, direct experimental vibration testing was conducted. The primary instruments and software employed for this purpose are summarized in Table 2.

Force Hammer: The force hammer applies a transient impact to a structure, and the structural response is typically measured by an attached accelerometer or a non-contact laser vibrometer. This measured response, when processed with an FFT analyzer, enables the derivation of the structure’s Frequency Response Function (FRF) and its operational mode shapes. The key specifications of the B&K 8210 model force hammer employed in this study are shown in Table 3:

**Table 3:** Force hammer parameters.

Specification	Value
Technology	CCLD
Sensitivity	0.225 mV/N
Range	22,240 N
Maximum Force	35,584 N
Head Mass	5448 g
Operating Temperature Range	−73 °C to 121 °C
Total Length	900 mm
Handle Material	Hardwood
Housing Material	Stainless Steel
Electrical Connector	BNC

Triaxial Accelerometer: This sensor enables simultaneous vibration measurement along three mutually perpendicular axes, facilitating comprehensive analysis of the entire structure. Each unit incorporates three independent, orthogonally oriented sensing elements. It features a multi-pin connector with independent cables for each axis, allowing separate output of the X, Y, and Z acceleration signals. The specifications of the employed PCB 356A16 model are shown in Table 4:

**Table 4:** Triaxial accelerometer parameters.

Specification	Value
Sensitivity	100 mV/g
Range	±50 g pk
Resolution	0.0001 g rms
Frequency Response (±5%)	0.5 to 4.5 kHz
Resonant Frequency	≥25 kHz
Temperature Range	−54 °C to +80 °C
Sensing Element	Ceramic/Shear
Output Connector	1/4-28 4-pin

Data Acquisition System: A Siemens LMS Software data acquisition system, widely employed in noise, vibration, and fatigue durability testing, was used. The key parameters of the utilized LMS SCADAS model are shown in Table 5:

**Table 5:** Data acquisition system parameters.

Specification	Value
Sampling Rate	204.8 kHz/channel, 24-bit A/D conversion
Bandwidth	92 kHz (independent of the number of channels)
Input Range	±316 mV to ±10 V, supports ICP sensor powering
Dynamic Range	>170 dB, phase matching accuracy 0.3° @ 10 kHz

Modal Analysis Software: The LMS Test.Lab 14A software suite was employed for this study. It is predominantly utilized for simulation and analysis in vibration and acoustic testing. A key feature of this system is its high-speed, multi-channel data acquisition capability, which facilitates test execution, data analysis, and the automatic generation of electronic reports. Its comprehensive functionality encompasses multi-channel synchronous sampling with real-time data transmission, digital filtering and spectral analysis, modal analysis and fatigue characteristic assessment, dynamic balance monitoring and shaft centerline orbit analysis, as well as noise and vibration measurement.

Controlled frequency and amplitude vibrations were introduced using dedicated excitation devices. In accordance with vibration transmission models, external excitation encompasses both mechanical (solid-borne) and acoustic (air-borne) sources.

Common mechanical excitation methods include force hammers and vibration motors. Force hammers, when integrated with test systems, generate transient impacts suitable for modal analysis of small components; however, their application to large equipment is challenging. In contrast, vibration motors produce steady periodic vibrations, making them suitable as controlled disturbance sources.

For acoustic excitation, loudspeakers (acoustic transducers) capable of generating specific-frequency sound waves were utilized, enabling the output of high-frequency pure tones.

**Figure 5:**
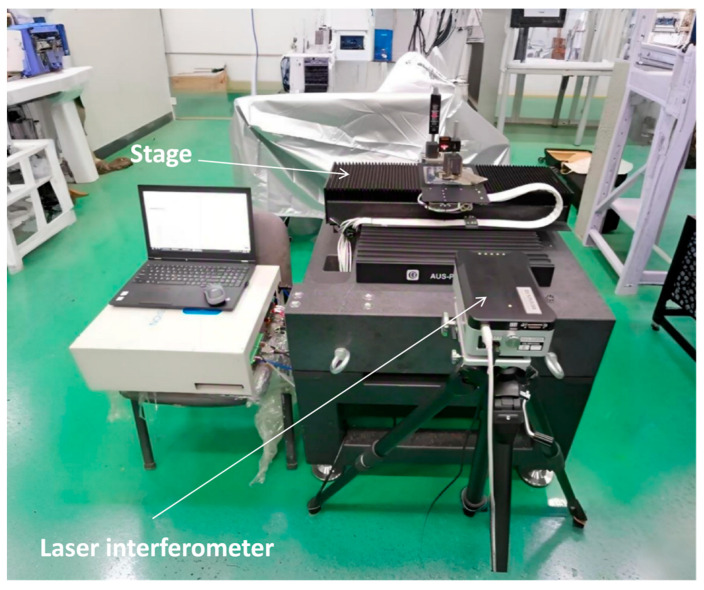
Atmospheric-side test platform of the stage.

A custom-developed stage test platform (shown in Figure 5) was utilized for two primary objectives: (1) to evaluate the positioning accuracy and motion control performance of the platform itself, and (2) to conduct vibration-related experiments and analysis on the EBI equipment.

For high-precision displacement measurement, a Renishaw RLU20 series laser interferometer served as the positioning device. It employs a He-Ne laser source transmitted via optical fiber to mitigate environmental interference. This system is particularly well-suited for vacuum and high-precision measurement applications, providing reliable displacement feedback essential for nanometer-level vibration compensation. Its specified performance—positioning accuracy of <1 nm and stability within ±20 ppb over 8 h—readily meets the requirements for both testing and compensation.

### 3.2. Compensation Scheme Design

The developed electron beam positioning compensation system operates on a closed-loop control architecture, which comprises three key components: sensors, a control unit, and actuators. The sensor suite primarily incorporates laser interferometers for displacement measurement, augmented with temperature sensors for environmental compensation. The actuators consist of electron beam deflectors and their associated high-speed drive modules. Serving as the system core, the control unit integrates a compensation module and a waveform generator module. This unit processes the sensor feedback in real time to calculate the positional error and subsequently generates the corresponding drive signals. By dynamically adjusting the line scan based on these signals, the system achieves precise positioning compensation.

**Figure 6:**
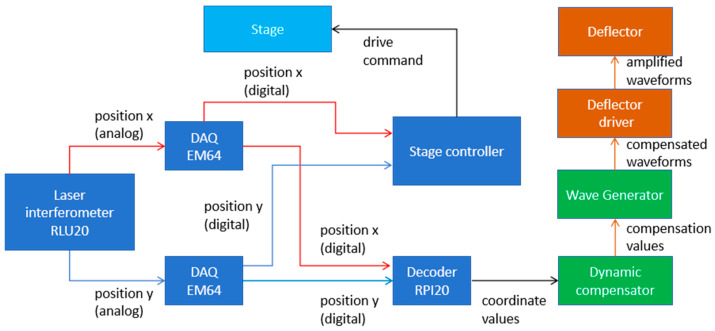
Block diagram of the electron beam positioning compensation system.

The overall architecture of this system is depicted in Figure 6.

The stage displacement in the X and Y directions was measured using a laser interferometer system (Renishaw RLU20, precision ≈ 0.1 nm). This system operates by detecting variations in the optical path length between reference mirrors and measurement mirrors mounted on the stage.

The positional data acquired by the interferometer is first digitized by a data acquisition device (DAQ EM64) and then decoded into coordinate readings by a dedicated decoder (RPI20). Subsequently, the dynamic compensation module calculates the deviation between the actual and expected stage positions.

Based on this deviation, the module computes the required compensation values. These values are fed to a waveform generator, which synthesizes the corresponding compensated scan waveforms. Finally, these waveforms drive the electron beam deflector via a high-voltage amplifier, generating a scanning electric field that adjusts the beam position on the sample in real time, thereby completing the closed-loop compensation.

### 3.3. Compensation Module Development

#### 3.3.1. Compensation Circuit Module Development

The compensation device centers on the real-time acquisition of positioning data and the high-speed generation of compensation waveforms. To achieve engineering efficiency, the control module hardware was custom-developed based on a standard architecture. Specifically, an EM64 board acquires the raw laser interferometer signal. Following acquisition, the signal is subsequently decoded into digital signals using an RPI20 module, which is compatible with the RLU series interferometer. To maximize signal transmission/calculation efficiency and real-time performance, the signal path length was minimized by modifying the existing waveform generator module to integrate the electron beam compensation board directly.

#### 3.3.2. Hardware Module Performance Characterization

Key performance indicators (KPIs) for the compensation hardware module encompass power stability, waveform linearity, rise time, and overall system delay. All major KPIs were verified through comprehensive testing to meet the design requirements. Time-domain analysis of the output waveforms, conducted using oscilloscopes, confirmed compliance with specifications for linearity, amplitude, and other critical characteristics. Furthermore, analysis of the waveform data characterized the system delay, which was measured to be less than 5 ms—a value that readily supports the compensation of jitter at frequencies below 200 Hz.

#### 3.3.3. Control Logic Software and Algorithm Development

The control software and algorithms are critical for determining system precision, flexibility, usability, and functional extensibility. The primary software manages the parameters output by the compensation module. Control parameters are sent from the host PC to the main control module, which becomes active during acquisition and calibration sequences. These parameters include key settings for electron beam image acquisition, such as pixel, line, and frame average counts; scan point offsets and sampling delays in both the column and line directions; blanker signal output delay; and scan mode, direction, and image angle.

To minimize communication latency and enhance control efficiency, the core compensation value calculation is implemented as firmware within the module’s embedded system. During debugging, the software interface provides tools for the visual confirmation of delay compensation effects on images and for quantitative analysis.

#### 3.3.4. Image Verification Processing and Auxiliary Preprocessing Algorithms

To verify the effectiveness of vibration compensation, processed images must be acquired and subjected to feature analysis. While jitter can often be identified visually or with simple frequency-domain algorithms, accurate evaluation requires the use of high-contrast, stable image features such as particles or edges. Feature extraction algorithms (e.g., SIFT, ORB, Harris corner detection) are employed to obtain coordinate sequences of these features. Analysis focuses on row offsets in the slow-scan direction to evaluate the line scan compensation.

**Figure 7:**
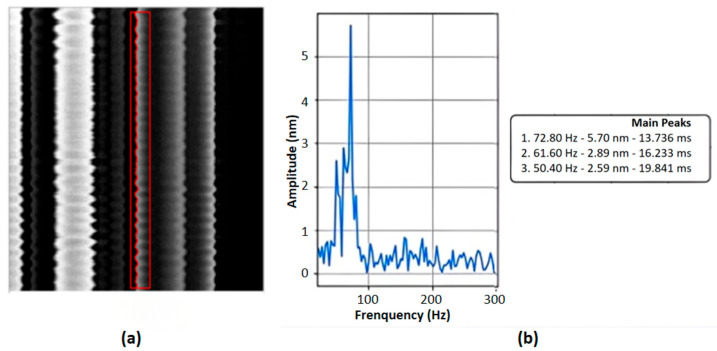
(**a**) Image obtained by FFT processing of the electron beam line-scan image. (**b**) Edge analysis based on line scan images yields the frequency spectrum.

Furthermore, the displacement sequence of these feature points can undergo Fast Fourier Transform (FFT). Peaks in the resulting spectrum correspond to the dominant frequency components of the jitter, with their amplitudes (requiring calibration) providing a quantitative measure of vibration intensity. Figure 7a shows the electron beam line scanning image. The red box indicates the area requiring image verification analysis, and the red line marks the actual image jitter. This approach is exemplified in Figure 7b, where edge analysis applied to line scan images yields the corresponding frequency spectrum.

**Figure 8:**
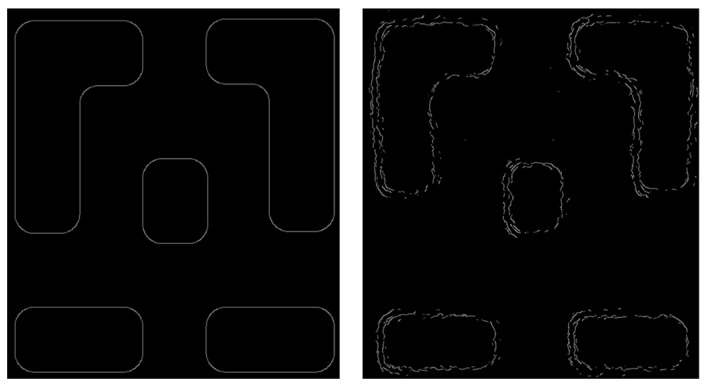
(**Left**): Ideal boundary pattern. (**Right**): SEM boundary image.

For images containing well-defined edges, edge extraction algorithms can be applied. Algorithms such as the Canny detector are used to extract the target edges. The coordinate deviations of these edge points from an ideal straight reference—caused by vibrational jitter—are then computed. This data is subsequently analyzed using a combination of curve-fitting methods and Fast Fourier Transform (FFT). The ideal pattern boundary alongside the corresponding boundary extracted from the SEM image using the Canny algorithm are presented for comparison in Figure 8.

In instances where the vibration amplitude is low or the SEM image exhibits a limited signal-to-noise ratio (SNR), single image frames may not accurately capture the vibration characteristics. In such cases, image preprocessing algorithms are essential for reliable verification. These algorithms perform operations such as noise removal and image alignment. Testing confirmed that applying band-pass filtering prior to alignment proved particularly effective.

1. Band-pass Filtering

**Figure 9:**
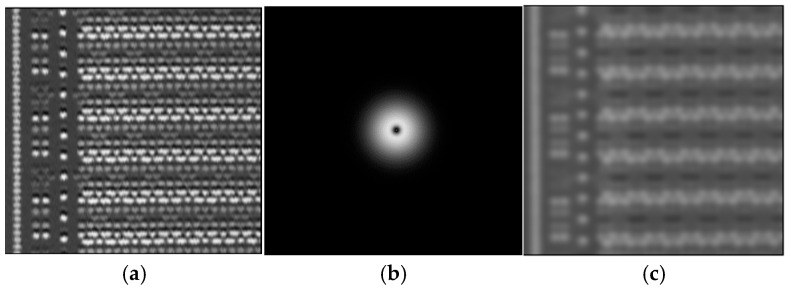
Band-pass filtering. (**a**): Original image; (**b**): Frequency spectrum image; (**c**): Filtered image.

A band-pass filter selectively transmits signals within a specific frequency range while attenuating components outside this band. When applied before image alignment, it removes both high- and low-frequency signals—effectively suppressing noise and certain non-essential image information—while preserving the majority of edge information. This preprocessing step significantly enhances alignment accuracy. The process is demonstrated in Figure 9: (a) the original image, (b) its frequency spectrum, and (c) the filtered result. The filter is configured to retain signals within the 5–40 frequency band (visible as a ring near the center in (b)). A comparison between (a) and (c) reveals that while the filtered image sacrifices some original signal content, it successfully eliminates most noise, thereby substantially improving the subsequent alignment process.

2. Image Alignment Method

Image alignment is a critical prerequisite for multi-frame averaging, a common technique employed for noise reduction. If sequential image frames possess positional deviations, misalignment occurs, which compromises the averaging process. To address this, an effective alignment method is proposed.

**Figure 10:**
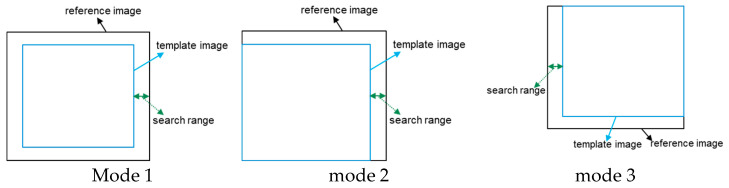
Image alignment mode.

The core of this method involves three distinct alignment modes, which are defined by the strategy used to crop the template image from the original full frame. These modes are schematically illustrated in Figure 10.

The proposed alignment method operates as follows. It initially employs Mode 1, calculating the inter-image offset using the Normalized Cross-Correlation (NCC) algorithm. A computed alignment score determines whether the alignment is successful. If this initial attempt fails, the vector sum conservation rule is applied to refine the offset. Should misalignment persist, the method sequentially switches to Mode 2 and, if necessary, Mode 3.

To enhance accuracy, sub-pixel offset calculation is performed, utilizing both image similarity and the conservation of offset vector sum as dual criteria. The three distinct template-cropping strategies inherent to these modes substantially lower the probability of misalignment, particularly for images with weak periodic patterns. Notably, suboptimal compensation can be identified when misalignment occurs during this process. This observed misalignment can then serve as an inverse indicator of the residual deviation, providing feedback to correct the compensation parameters.

## 4. Experimental Verification of Vibration Compensation Scheme

### 4.1. Experimental Verification Data

As previously discussed, for EBI equipment such as the i525 prototype, the proposed vibration compensation system specifically targets mechanical vibrations below 200 Hz, as these frequencies are most detrimental to image quality and metrology precision.

To verify the compensation effectiveness within this frequency band and to isolate contributing factors, the experimental campaign encompassed four key tests: (i) compensation angle testing and optimal angle calibration; (ii) scan angle correlation testing; (iii) frequency response testing across different frequencies; and (iv) stage position correlation testing.

The effectiveness of the compensation was evaluated through analysis of the resulting images. The outcomes of these tests are detailed in the following subsections.

#### 4.1.1. Compensation Angle Testing and Optimal Angle Calibration

The electron beam vibration compensation operates by generating a corrective vector displacement via the deflection field. However, the actual direction of this compensation vector can deviate from the intended path due to several practical factors. These include inherent errors in the electrostatic deflector (e.g., leveling and rotational misalignment), non-uniformities in the objective lens magnetic field, voltage output imperfections (ripple and offset) in the drive circuits, and installation inaccuracies in the positioning sensors themselves, which affect the accuracy of vibration detection.

In practical imaging, relative angular misalignments between the electron optical system, the main chamber, and the stage can introduce an angular mismatch between the intended compensation displacement direction and the actual vibration direction. Therefore, identifying the optimal compensation angle—where vibration cancellation is most effective—and calibrating this angular deviation are essential steps when enabling the compensation system.

For this test, the reference angle (0°) was defined relative to the stage’s X-direction. The compensation angle was then systematically varied to determine its optimal value. First, a preset deflection-objective excitation parameter table was calibrated to minimize systematic errors. A coarse adjustment was performed across the full angular range (e.g., in 45° steps) to identify intervals yielding better compensation performance. Subsequently, the step size was progressively reduced for fine adjustment until the compensation performance met the specified requirements.

**Figure 11:**
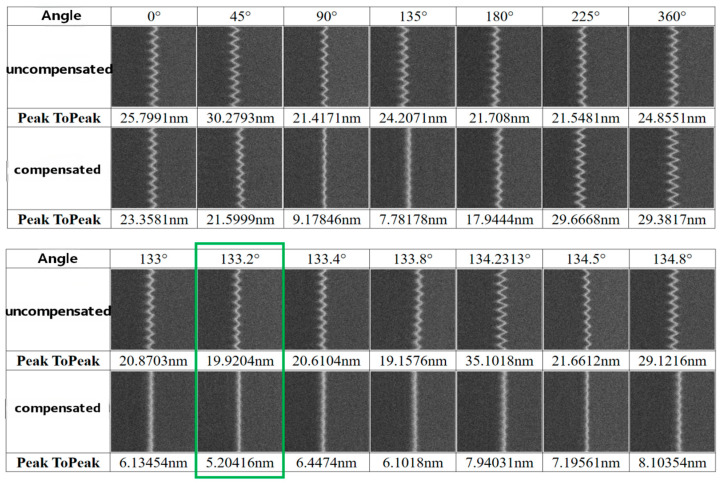
Test results of different angle compensation cases (selected test images and data).

For example, as presented in Figure 11, under an 80 Hz external vibration excitation (with the vibrator aligned along the stage diagonal, resulting in an excitation angle of approximately 135 ± 2.5°), the uncompensated jitter amplitude was approximately 20–30 nm (peak-to-peak). After enabling the compensation system, preliminary tests identified 135° as the preliminary optimal angle. Subsequently, a fine-adjustment process employing smaller angular steps (0.2°) was conducted, which refined the optimal compensation angle to 133.2°, as indicated by the green box in Figure 11. This precise calibration reduced the residual jitter to 5.2 nm (peak-to-peak).

This result confirms that the experimentally determined optimal compensation angle (133.2°) falls well within the estimated error range (±2.5°) of the applied excitation angle (~135°).

Under identical experimental conditions and using 135° as the baseline compensation angle, tests were repeated across various excitation frequencies to determine whether the optimal angle deviation exhibits frequency dependence. It was hypothesized that this deviation would be largely insensitive to the vibration frequency. A significant variation in the optimized angle with frequency would necessitate a more complex compensation system architecture.

**Figure 12:**
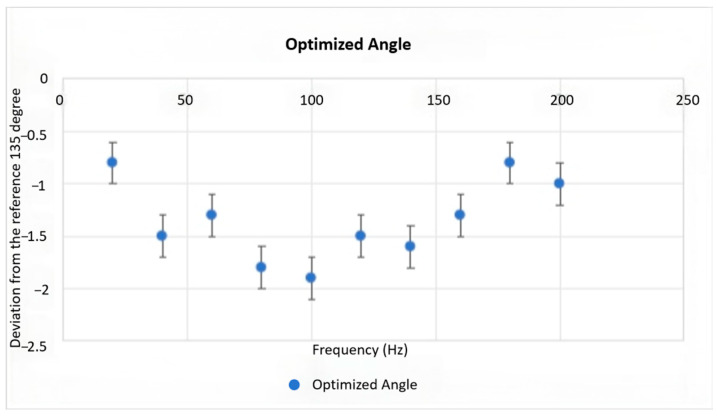
External vibration excitation at different frequencies.

As shown in Figure 12, the deviation of the optimized angle relative to the 135° baseline, measured under different excitation frequencies, consistently falls within the ±2.5° error margin. This result confirms the initial hypothesis, demonstrating a low correlation between the optimal compensation angle and the excitation frequency. Consequently, the observed insensitivity is deemed acceptable for practical inspection requirements.

#### 4.1.2. Scan Angle Correlation Test

Based on the results from the aforementioned tests, the optimal compensation angle was determined and set as a fixed input parameter for the system. To validate this angle calibration and to assess the impact of the beam scanning direction on compensation performance, the jitter compensation effectiveness was evaluated across a range of scan angles. Here, the scan rotation angle is defined as the relative angle between the electron beam’s fast-scan direction (X) and the stage’s primary axis of motion (X-axis).

**Figure 13:**
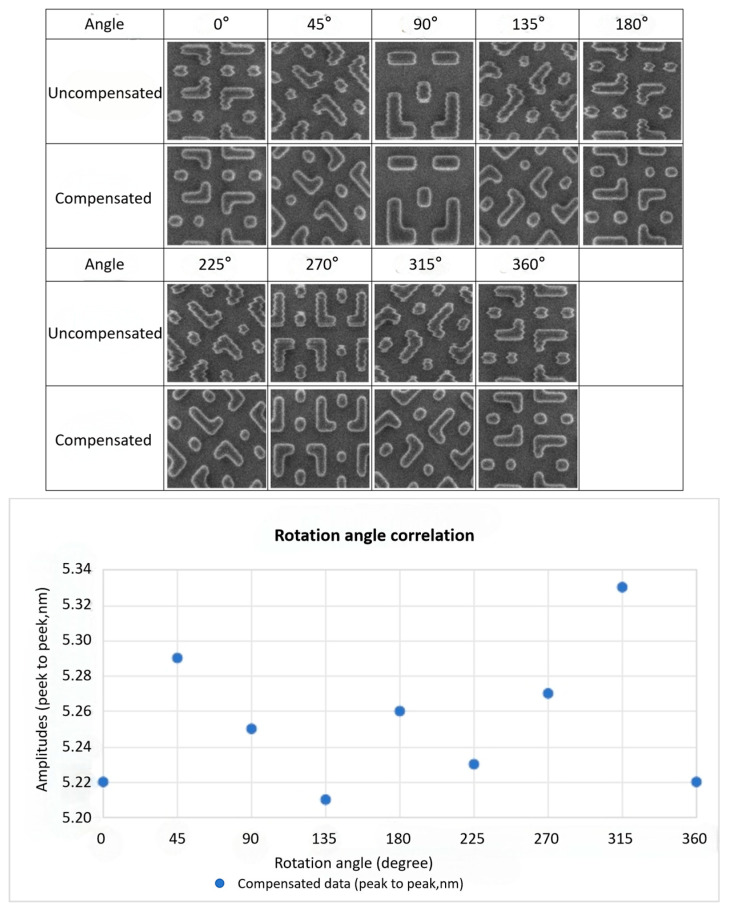
After angle compensation correction, changing the scan angle has no visible effect on the compensation performance.

Figure 13 demonstrates that after the angle calibration procedure, the compensation effectiveness remains consistent across different scan angles. This result confirms the validity of the calibration.

Statistical analysis of the calibration results across different compensation angles yields an average compensated amplitude error within 0.15 nm. Furthermore, the corresponding 3-sigma value, derived from multiple measurements, is 0.11 nm. These values meet the precision requirements for general inspection and metrology applications.

#### 4.1.3. Different Frequency Compensation Test

To explicitly describe the characteristic frequencies under key conditions, Table 6 summarizes the main vibration frequency components identified through experiments for various typical operating and test scenarios. This provides clear frequency targets for the subsequent verification and analysis of the compensation performance.

Validating the system’s compensation capability across different vibration frequencies is central to its performance and represents a critical technical benchmark. To this end, the dedicated vibration exciters mentioned previously were employed to generate controlled vibrations across the spectrum below 200 Hz.

**Figure 14:**
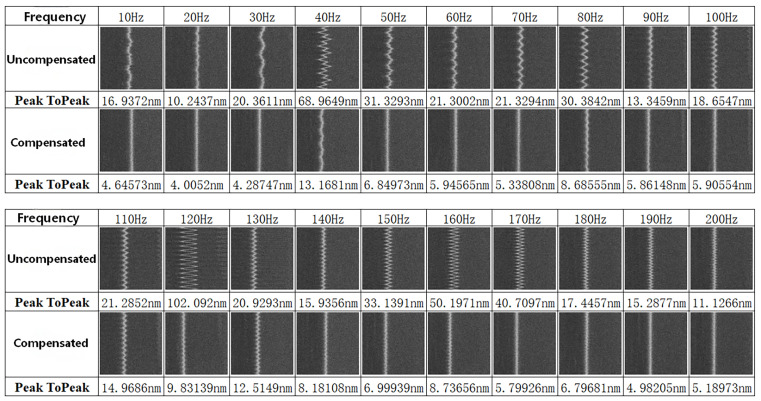
Compensation effectiveness under vibrations of different frequencies from 10 Hz to 200 Hz.

As demonstrated in Figure 14, the compensation system effectively attenuates a broad range of vibrations below 200 Hz.

**Table 6:** Summary of main characteristic frequencies under different experimental and operating conditions.

Test Condition	Main Characteristic Frequencies (Hz)	Source and Analysis
Background Noise (Equipment standby, shield door closed)	~5–10, ~50–60	Primarily originates from environmental vibrations (e.g., building structure, HVAC systems) transmitted through the foundation, manifesting as broadband, low-energy disturbances.
External Single-Frequency Excitation (Actively applied during experiments)	10, 20, 40, 80, 120, 160, 200	Specific frequency points generated by a shaker or loudspeaker to verify the system’s compensation capability, covering the target compensation bandwidth (≤200 Hz).
System Resonance Frequencies	~40, ~120, ~160	The first few natural frequencies of the equipment’s mechanical structure identified through modal analysis (impact hammer test). Vibration response at these frequencies is significantly amplified when excited (as shown by the peaks in Figure 15).
Internal Component Operation (Example: Turbomolecular Pump)	~400–800 (e.g., 600 Hz)	Harmonic vibration caused by rotor imbalance in high-speed vacuum turbomolecular pumps, belonging to mid-frequency sources. As the compensation scheme primarily targets frequencies below 200 Hz, vibrations in this band rely on passive suppression via isolation design.

**Figure 15:**
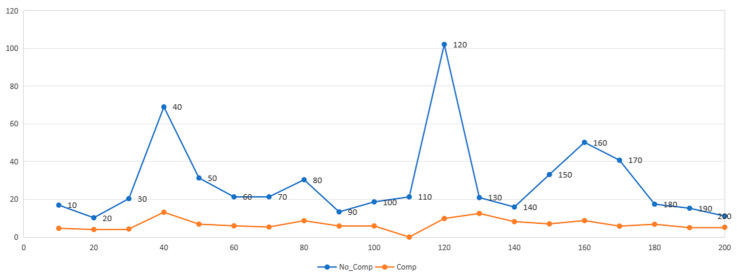
Compensation effectiveness of the system under external vibration excitation at different frequencies.

The system demonstrated effective compensation for single-frequency mechanical vibrations below 200 Hz, with multiple tests (*n* > 5 per frequency band) resulting in a significant reduction in jitter. The averaged results from these single-frequency vibration compensation tests across the 10 Hz to 200 Hz range are summarized in Figure 15.

The single-frequency compensation tests confirm that the developed system effectively counteracts vibration-induced image displacement, achieving an average attenuation rate exceeding 85%. At the system’s characteristic resonance peaks (e.g., approximately 40 Hz, 120 Hz, and 160 Hz, as indicated by the blue curve in Figure 15), the average jitter attenuation reaches 85% and can be as high as 92%.

**Figure 16:**
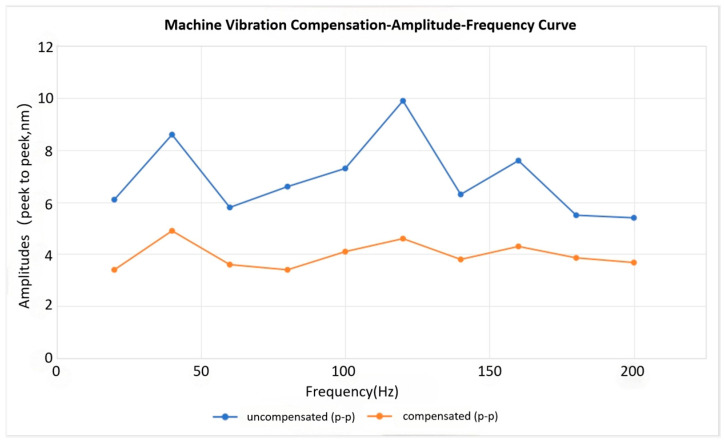
Amplitude-frequency curve of equipment vibration after compensation without externally introduced excitation.

Under actual operating conditions of EBI equipment, where vibration sources are mitigated by isolators and shielding doors, the vibrational energy impacting the image is typically lower than the direct excitation levels used in this study. Therefore, evaluating the compensation performance under such non-high-power excitation conditions is equally important. The corresponding amplitude-frequency curve obtained after vibration compensation, without any externally introduced excitation, is presented in Figure 16.

As shown in Figure 16, under conditions with no externally introduced excitation (and with the equipment’s shielding doors removed), the residual vibration—primarily originating from the vacuum system and environmental noise—exerts a relatively limited influence, with measured amplitudes typically ranging from 5 to 10 nm (peak-to-peak).

After applying the developed compensation system, the relative attenuation ratio under these conditions is notably lower (approximately 50–70%) compared to that achieved under direct experimental excitation. However, the final compensated amplitude is significantly reduced, achieving a stable residual jitter of only 4–5 nm (peak-to-peak).

#### 4.1.4. Stage Position Correlation Test

**Figure 17:**
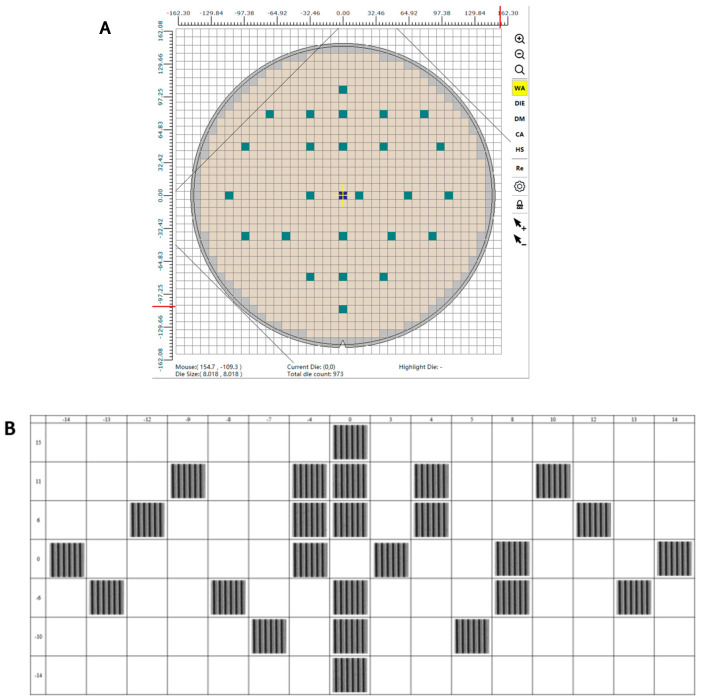
Validation of vibration compensation effectiveness at various positions on a 12-inch wafer stage.

The structural design and operational principles of the stage introduce the possibility that vibration behavior and the accuracy of mirror-based positioning measurements could vary with stage position, potentially influencing compensation performance. To investigate this potential correlation, a 12-inch wafer was placed on the stage, and measurements were conducted at a set of predefined points. Twenty-five such points distributed across the wafer were selected for compensation effectiveness verification, as illustrated in Figure 17A. Analysis of the post-compensation images obtained at each point (Figure 17B) confirms that compensation performance is consistent across the stage, effectively ruling out any significant stage-position-dependent correlation.

The experimental tests detailed above successfully verify the practical effectiveness of the vibration compensation system on the equipment. For vibrations below 200 Hz, the system provides substantial compensation, suppressing mechanical vibration-induced image jitter by over 75%. As the compensation performance is angle-dependent, initial angle calibration is essential. Once properly calibrated and enabled, the system’s effectiveness shows low correlation with both the beam scan angle and the stage position, eliminating the need for additional positional compensation or correction.

### 4.2. Image-Based Compensation Effect Data Analysis

**Figure 18:**
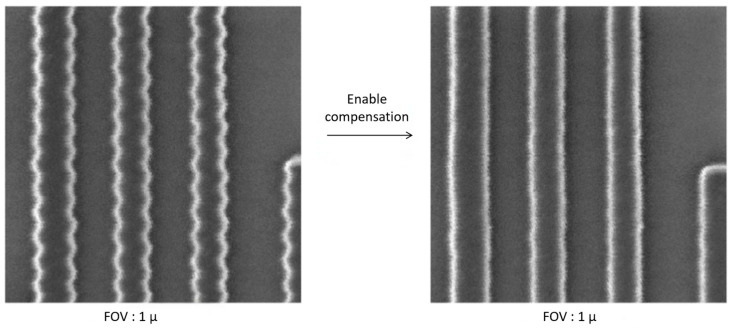
Compensation test results for introduced 70 Hz vibration at 1 μm FOV.

Building on the experimental verification, the feasibility of the compensation scheme is confirmed. A representative result is shown in Figure 18, where enabling vibration compensation under normal equipment operating conditions reduces the scanning jitter from 16 nm (peak-to-peak) to 4 nm (peak-to-peak). This level of performance meets the practical requirements for equipment operation.

In practical operation, equipment is often subject to simultaneous vibration interference from multiple frequency bands. Therefore, validating the compensation system’s efficacy under both single-frequency and composite multi-frequency conditions is critical for assessing its overall performance.

**Figure 19:**
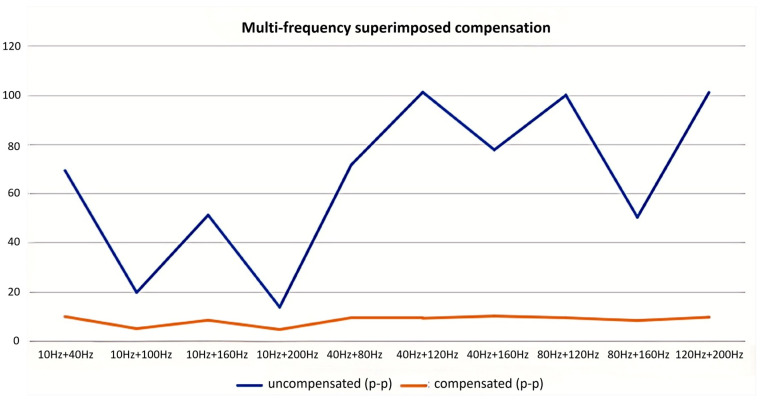
Compensation effectiveness under multi-frequency mixed vibration conditions.

To replicate this scenario, experiments involved superimposing excitation signals from different frequency bands and evaluating the resulting compensation effectiveness. Experimental results confirm that the proposed vibration compensation scheme functions effectively under multi-frequency excitation. As demonstrated in Figure 19, the system provides significant compensation when subjected to superimposed vibrations composed of frequency components within the 10 Hz to 200 Hz band.

Quantitative results from the multi-frequency excitation tests are summarized in Figure 19. Following multi-frequency excitation, the uncompensated vibration amplitude ranged from 20 to 100 nm peak-to-peak (±10 to ±50 nm). After enabling the compensation system, the average compensated amplitude across the 10–200 Hz band was reduced to 8.23 nm *p-p* (±4.11 nm). At frequencies away from system resonances, the amplitude was further reduced to 4.10 nm *p-p* (±2.05 nm).

Integrating these results with the earlier single-frequency tests confirms that the vibration compensation system provides significant attenuation—exceeding 50%—for both single-frequency and composite multi-frequency vibrations.

**Figure 20:**
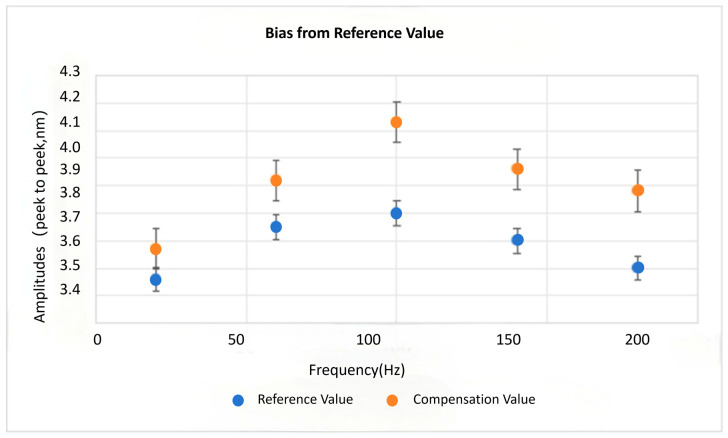
Deviation of the reference value in the compensation system.

To establish a performance baseline under optimal conditions (with shielding doors and isolators effectively engaged), the minimum image vibration amplitude at non-characteristic frequencies was measured. The average baseline value over 10 measurements was 3.68 nm *p-p* (±1.84 nm). When the compensation system was active, the average deviation from this baseline was merely 0.23 nm. The distribution of deviations across frequency points is detailed in Figure 20.

The deviation data confirms that the compensation system’s residual error is maintained below 10% of the established vibration baseline, thereby satisfying the requirements for metrology and inspection applications.

### 4.3. Repeatability Test Data

In engineering applications of electron beam metrology and inspection, repeatability precision is a critical performance metric. It is therefore essential to ensure that the vibration compensation system does not introduce additional errors that could compromise imaging accuracy or sensitivity.

To further validate the long-term stability of the compensation, a series of repeatability tests were conducted. The test protocol involved performing 10 consecutive measurements on a standard pattern (e.g., a dense line pattern) and calculating the 3-sigma value as the measure of repeatability precision. The corresponding results are presented in Table 7. The average repeatability precision was determined to be 0.27 nm, which is below the standard benchmark of 0.35 nm for routine testing. Thus, the compensation system’s repeatability precision is confirmed to meet the necessary specifications.

**Figure 21:**
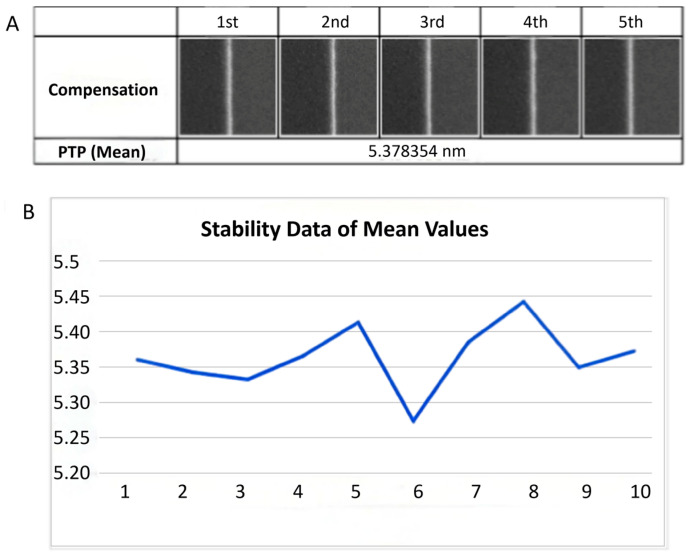
Repeatability test data at different locations (mean) (**A**): First set of test values; (**B**): 10 interval test data points (with 12 h intervals).

To assess the long-term stability of the compensation system, measurements were conducted on similar samples at extended time intervals. The experimental approach involved recording the mean compensated amplitude at multiple measurement points over time. Specifically, the test protocol consisted of performing amplitude measurements on a set of similar samples (5–10) at different times, with intervals of 8 to 12 h between measurements, followed by statistical analysis of the collected data. The resulting long-term stability data are presented in Figure 21.

The analysis shows that the average relative error over a 12 h period remains below 0.85%. This result meets the stringent stability requirement of <1.0% for practical production line equipment, thereby confirming the system’s suitability for sustained industrial operation.

### 4.4. Error Source Analysis

It is important to note that the presented vibration compensation system cannot ensure the complete elimination of all image jitter. Attention must be given to other interfering factors, as well as the impact of various parameter settings on the overall compensation efficacy. The primary sources of residual error can be categorized as follows:

1. High-Frequency Vibration and Electromagnetic Interference (EMI)

**Figure 22:**
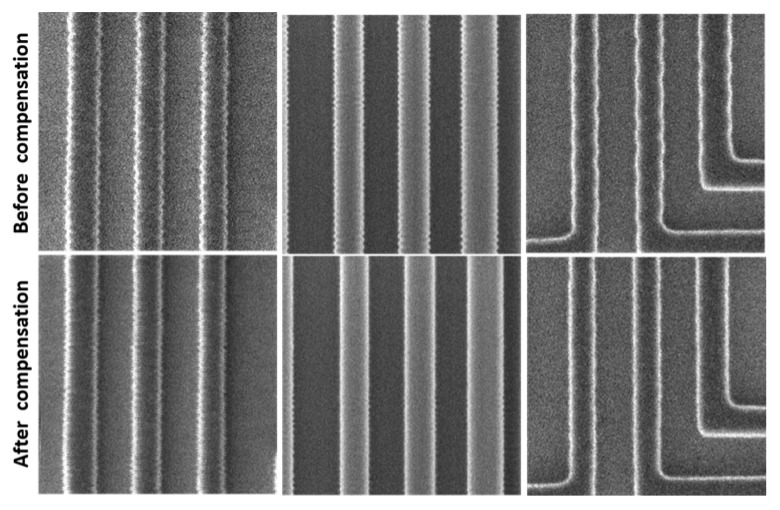
After vibration compensation, low-frequency vibration signals are suppressed under three interference conditions—(**left**) (100 Hz), (**middle**) (70 Hz & 100 Hz), (**right**) (50 Hz)—though high-frequency electromagnetic interference persists.

As demonstrated in Figure 22, enabling the vibration compensation leads to significant improvement in low-frequency image jitter. However, Fast Fourier Transform (FFT) analysis reveals that residual jitter components originate from frequencies above 300 Hz. This high-frequency interference was confirmed as external electromagnetic interference (EMI) using a dedicated Spicer electromagnetic measurement device, a condition exacerbated by the removal of the equipment’s shielding door. Since the developed compensation system operates on a positioning-based principle, it is inherently unable to compensate for this type of high-frequency EMI.

2. Phase and Delay Calibration Parameters

A critical consideration is that the phase and delay compensation parameters require independent calibration for each specific system. Failure to perform this system-specific calibration can introduce additional compensation errors, thereby compromising the effectiveness of the vibration suppression or, in severe cases, intensifying the image jitter.

**Figure 23:**
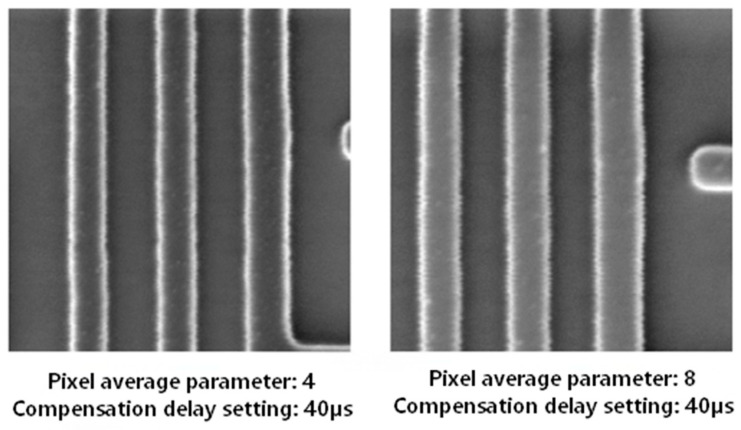
After increasing the compensation signal averaging count, failure to adjust the compensation delay parameter leads to phase difference in the compensation signal, worsening compensation effectiveness.

As illustrated in Figure 23, increasing the scan averaging count extends the single-line scan time (comparing the right panel to the left). If the corresponding compensation delay parameter is not recalibrated, a phase misalignment is introduced in the compensation signal, which directly compromises its effectiveness.

### 4.5. Comparison with Classical Methods

A comparative analysis was conducted between the test data of the proposed scheme and traditional methods (the classical method), with the specific performance and characteristics summarized in Table 8. Early classical studies (e.g., Jung et al. [2]) primarily addressed image serration distortion caused by internal pump vibrations in Scanning Electron Microscopes (SEMs), employing a phase-inversion compensation method based on image frequency analysis. This method could reduce the amplitude of a 170 Hz vibration signal from 7 mV to 2.25 mV and decrease the pixel distortion at image edges from an average of 4–6 pixels to 2–4 pixels, preliminarily validating the effectiveness of vibration compensation via signal injection.

In contrast, the proposed scheme establishes a closed-loop dynamic compensation system based on real-time laser positioning to address more complex broadband vibration environments. Its testing framework is more systematic. The results indicate that the system achieves an average attenuation exceeding 85% for single-frequency vibrations within the 10–200 Hz range and can suppress the peak-to-peak displacement amplitude to 8.23 nm for multi-frequency composite vibrations. More importantly, this study provides quantified metrics directly relevant to industrial application reliability, including a repositioning accuracy (3σ) of 0.27 nm and a long-term stability error of less than 0.85%.

In summary, the evolution evident from the test data shows that research in this field has progressed from principle-based methods aimed at improving specific image quality to systematic engineering solutions targeting nanometric manufacturing precision and production-line stability. The proposed scheme demonstrates significant improvements in compensation bandwidth, accuracy level, repeatability, and long-term stability, marking the entry of vibration compensation technology into a matured application stage capable of meeting the demands of high-end manufacturing.

## 5. Conclusions

Electron beam metrology and inspection equipment, being high-precision instruments operating at the nanometer scale, are critically susceptible to vibrational disturbances that degrade performance. These vibrations, originating from both external environmental sources (solid-borne and acoustic) and internal equipment operations (stage motion, valve actuation, etc.), induce relative displacement between the wafer and the electron beam. This leads to image artifacts such as positional errors and blurring, posing a significant challenge to measurement accuracy.

To address this, a closed-loop vibration compensation system was developed. The technical implementation comprised: (1) a laser interferometer serving as a nanometer-precision positioning sensor; (2) electron beam deflectors with their drive modules as actuators (featuring quadrupole electrostatic deflectors capable of >10 kHz compensation in the fast-scan direction); and (3) custom-developed signal processing boards integrated with a waveform generator for real-time data processing and compensation signal synthesis. This integrated design enhanced signal transmission efficiency and minimized loss.

Through dedicated hardware and software development followed by comprehensive performance testing, the system’s core components—including its control algorithms—were successfully implemented and validated. The supporting software underwent modular testing, confirming its capability for multi-parameter and multi-degree-of-freedom adjustment. Additionally, verification tools incorporating filter-based noise suppression, multi-frame image alignment, Canny edge extraction, and FFT-based jitter analysis were established.

System integration and calibration on actual equipment demonstrated the efficacy of the proposed technology. The compensation system operates effectively against both single-frequency and multi-frequency vibrations below 200 Hz, achieving an average attenuation of 50% to over 90%. Key performance metrics include a compensated amplitude deviation not exceeding 0.25 nm relative to the quiet background, a repeatability precision below 0.3 nm, and a long-term stability error under 0.9%. Once calibrated, the optimal compensation angle for vibration in a specific direction (e.g., 135°) remains effective across different vibration frequencies. These results verify that the developed system meets the stringent practical requirements for vibration compensation amplitude and precision, confirming its readiness for industrial application.

## Figures and Tables

**Table 1:** Typical Scanning parameters for electron beam inspection/metrology equipment.

	Pixel Scan Time	X Pixel Count	Y Pixel Count	Line Scan Time	Line Frequency	Frame Scan Time	Frame Frequency
1	100 ns	1024	1024	~100 μs	10 kHz	~100 ms	10 Hz
2	100 ns	512	512	~50 μs	20 kHz	~40 ms	25 Hz
3	30 ns	512	512	~15 μs	67 kHz	~7.5 ms	133 Hz

**Table 2:** Instruments for vibration characterization and testing.

Name	Manufacturer	Model	Purpose
Force Hammer	B&K (Denmark)	B&K 8210	Generate excitation signal
Triaxial Accelerometer	PCB (Buffalo, New York, USA)	356A16	Measure response signal
Data Acquisition System	LMS (Belgium)	SCADAS	Analog signal acquisition
Modal Analysis Software	LMS (Belgium)	Test.Lab14A	Modeling, transfer function calculation, modal analysis

**Table 7:** The 10-repeat test data (3-sigma error).

Die No.	1	2	3	4	5	6	7	8	9	10
1st	69.93399	70.408768	70.254402	69.721664	69.340111	70.80809	71.187073	70.743996	69.876892	70.912086
2nd	69.709361	70.618812	70.236964	69.627896	69.41195	70.77251	71.243845	70.781119	69.675514	70.542021
3rd	69.81854	70.354759	70.172689	69.757905	69.488482	70.710685	71.044102	70.672742	69.941589	70.665439
4th	69.713111	70.54869	70.182625	69.747808	69.086727	70.768122	71.318899	70.714626	69.789176	70.58172
5th	69.820795	70.346213	70.082698	69.644434	69.306562	70.873747	71.081306	70.682223	69.762297	70.661605
6th	69.98045	70.369788	70.206007	69.752692	69.256949	70.736299	71.207987	70.72091	69.794974	70.70715
7th	69.705184	70.538771	70.178781	69.44778	69.318177	70.744214	71.197178	70.821068	69.856436	70.640963
8th	69.809389	70.50833	70.185612	69.530365	69.372286	70.820702	71.058195	70.563612	69.795339	70.768921
9th	69.824558	70.593481	70.288787	69.744191	69.266017	70.831592	71.345947	70.718702	69.782773	70.64411
10th	69.834143	70.344198	70.194398	69.845812	69.445981	70.723724	71.127146	70.812504	69.847806	70.749477
3σ	0.275079	0.327933	0.16647	0.359938	0.341278	0.158885	0.312827	0.226126	0.217489	0.314411

**Table 8:** Comparison of vibration compensation test data.

Comparison Dimension	Classical Method	Proposed Scheme
Primary Vibration Source	Internal equipment pumps (e.g., rotary pump, turbopump)	Combined internal/external vibrations (environmental, equipment operation)
Target Frequency	Specific frequencies (e.g., 30, 60, 120, 170 Hz)	Broadband (≤200 Hz)
Core Method	Phase-inversion signal injection (based on image analysis)	Closed-loop dynamic compensation (based on real-time laser positioning)
Key Performance Metrics	170 Hz signal amplitude: 7 mV → 2.25 mV Pixel distortion: 4–6 pixels → 2–4 pixels	Average attenuation rate: >85% (single frequency) Amplitude after multi-frequency vibration: ~8.23 nm (*p-p*) Repositioning accuracy (3σ): 0.27 nm Long-term stability error: <0.85%
Test Systematics	Validation of a specific method’s effectiveness for a single problem	Multi-dimensional validation (angle, frequency, position); repeatability and long-term stability assessment
Technology Stage	Proof of concept & early-stage solution	Mature, industrial-grade systematic engineering solution

## Data Availability

The data presented in this study are available on request from the corresponding author.
